# Risky sexual behaviours among adolescent undergraduate students in Nigeria: does social context of early adolescence matter?

**DOI:** 10.11604/pamj.2020.37.188.22968

**Published:** 2020-10-28

**Authors:** Aloysius Odii, Chiemezie Scholastica Atama, Ijeoma Igwe, Ngozi Justina Idemili-Aronu, Nkechi Genevieve Onyeneho

**Affiliations:** 1 Department of Sociology and Anthropology, University of Nigeria, Nsukka, Nigeria

**Keywords:** Adolescents, social context, sexual behaviour, risky sexual behaviour, sex education

## Abstract

**Introduction:**

adolescent undergraduate students engage in sexual acts that put them at risk of contracting Sexually Transmitted Infections (STIs) and unwanted pregnancies. Even though the social context of early adolescence accounts for developmental changes in later phase of life, its impact on adolescents' risky sexual behaviour has not been extensively explored. In this study, we examined how the social context of early adolescence influenced adolescent's risky sexual behaviour in the university.

**Methods:**

qualitative data were collected from 24 adolescent undergraduate students of four universities in South-eastern Nigeria. Both males and females, within the age of 16-19 years were interviewed. The data were coded, managed with the use of Atlas.ti software and thematically analysed.

**Results:**

the findings indicated that risky sexual behaviours among adolescent undergraduate students are embedded in the quality of sex education by parents at early adolescence. As such, unprotected sex and multiple sexual partners were rampant among adolescents who were not exposed to quality sex education at early adolescence. Adolescents whose parents are religious and/or authoritative but did not teach sex education during early adolescence engaged in risky sexual behaviours in the university. Also, adolescent undergraduate students that were raised in rural areas indulged in unprotected sex because of limited access to sex education during early adolescence.

**Conclusion:**

social context of early adolescence means a lot for adolescents' sexual experience in later phase of life. When parents provide their children the right information about sex, it can protect them from risky sexual behaviours as they grow older.

## Introduction

Globally, adolescents´ population is estimated to be 1.2 billion [1] and the majority of them are sexually active [2]. Often, adolescents practice sex in unsafe conditions that expose them to various sexual and reproductive health problems [3,4]. The consequences of these are dire and sometimes contribute to premature deaths [5]. Nigeria is home to one-third of all adolescents in developing countries [5] and many of them are at risk of being infected with STIs, Human Immunodeficiency Virus (HIV) and other associated consequences of unsafe sex. In South-eastern Nigeria alone, 17% of adolescents have shown prevalence of STIs and HIV while 32% have had unsafe abortions [6]. Therefore, studies on adolescents´ sexual behaviours are important because promoting healthy behaviours and preventing risky sex among adolescents are important for the country´s future health [1] and the attainment of Sustainable Development Goals (SDG) of 2030.

Adolescents in tertiary institutions engage in high-risk sexual behaviours like unprotected intercourse and multiple sexual partners [7-9]. Undergraduates are known to be freer from the control of family and university authorities [10] and this freedom provides greater opportunity for risky sexual behaviours [11]. Although many students are aware that consistent condom use prevents STIs and unwanted pregnancy, they continue to engage in unprotected sexual intercourse [12]. Commonly cited reasons for unprotected sex are that it interferes with pleasure during sex and that it was unnecessary if they trust their partners [13].

Social context or social environment has a role to play in fostering and enriching our understanding of adolescents´ sexual behaviours in the university. This is because they constitute the forces that shape people´s daily experiences and also, directly or indirectly affect their behaviour [14]. In contrast to studies that have examined the social context of risky sexual behaviours among adolescents [15,16], the current study examines how social context of early adolescence shapes adolescents´ risky sexual behaviours in the university. We believe that early adolescence stage is important because it is characterised by development of sex organs and other sexual characteristics that could be the source of anxiety, excitement, or pride [17]. Moreover, it is also a period when important sex education is required. We argue that since early life experiences often have long-lasting impact on behaviour in later phase of life or adulthood [18], several forces acting during early development can have intricate effects on behaviour, including sexual behaviour in later phase. Hence, explanations of adolescents´ risky sexual behaviour in the university should point to underlying forces in their early development.

The study is guided by the Developmental Contextual Framework (DCF) which holds that development is the associations between the developing individual and their complex and changing social and physical context [19]. One of the key themes to this perspective is that human development is rooted within social contexts or that it is through interactions with people that an individual´s characteristics have meaning and undergoes changes [20]. From this perspective, adolescents´ development does not occur in isolation but rely on contacts and interactions with those who make up the primary social contexts: family members, peer group, religious group etc. [21]. Activities and interaction with these contextual factors at early adolescence forms the social norms that guide behaviours in later phase of life [22]. Using a developmental contextual framework, this study aims to examine the link between social context of early adolescence and the perpetration of risky sexual behaviours among adolescents in the university.

## Methods

**Area of study:** the study was carried out in both the federal and state universities in Enugu and Ebonyi states, South-eastern Nigeria. The federal universities were the University of Nigeria, Nsukka, Enugu State (UNN) and Alex-Ekwueme Federal University of Ndufu-Alike, Ikwo Ebonyi State (AE-FUNAI). The state universities were Enugu State University of Science and Technology Enugu (ESUT) and Ebonyi State University (EBSU). UNN has three campuses: Nsukka, Enugu and Ituku Ozalla. AE-FUNAI has one campus. ESUT has two campuses: Agbani and Enugu while EBSU has four campuses: CAS, Presco, Ishieke and Permanent Site. For Universities with multiple campuses, the campus housing the administrative quarters were chosen: Nsukka campus was chosen for UNN, Agbani was chosen for ESUT while the Permanent Site was chosen for EBSU.

**Participants selection:** undergraduate students of UNN, EBSU, AE-FUNAI and EBSU within the ages of 16-19 were the respondents for the study. The start from 16 years was because most universities admit students only when they are 16 years old and above. Also, we focused on students in second years because they already had a year experience in their respective universities. A total of 24 in-depth interviews, involving 12 males and 12 females, were conducted for the study; in each university, six interviews involving three males and three females were conducted. The inclusion criteria were that the participants are within the specified age of 16-19 years, must be in second year and must reside within or outside school hostels. Adolescents who met these criteria but reside with their parents were excluded from the study because we suspected that they do not exercise the same freedom as those who reside separate from their parents [10]. Participants were selected based on these criteria and also based on the willingness to participate in the study.

**Ethical consideration:** the study´s purpose and process were properly explained to the participants and they were assured of anonymity and confidentiality. They were given a choice to either participate or decline, those who agreed to participate signed the written consent form. Before the interview commenced, participants were informed that they can disregard any question and stop the interview whenever they feel like. Verbal consent was sought to record the interviews.

**Instrument and data collection:** the study made use of an in-depth interview (IDI) guide as the instrument for data collection. The IDI guide contained questions that examined the socio-contextual forces experienced at early adolescence and how it influenced risky sexual behaviours in the university. By social context, we refer to the social relationships, surroundings and cultural milieu within which participants functioned and interacted at early adolescent phase (or shortly before admission into the university). The guide sought the interaction with parents, peer group and religious body about sexual activities and how such interactions influenced risky sexual behaviours in the university. By risky sexual behaviours, we refer to unprotected sex and multiple sexual partners. The researchers facilitated the interview sessions and the note-taking sessions. A tape recorder was also used during the session to enable the recording of the interview. Throughout the research, all ethical procedures bordering on confidentiality, privacy, consent, etc. were followed. IDIs were held at the time and spot convenient for the participants. The interviews were conducted in English language and it lasted for an average, 45 minutes.

**Methods of data analyses:** data collected were transcribed by the researchers and managed with the use of Atlas.ti software. Before coding, the data were read multiple times to ensure complete understanding. The transcripts were coded using open coding whereby each unit of text was assigned a code(s). The code was organised in themes using axial coding by reorganizing and making connections among the various categories and subcategories raised. In many instances, we pulled phrases with contextual or special connotations as illustrative quotes to build up the conversation under each theme.

## Results

**Socio-demographic characteristics of respondents:** a total of 24 interviews, consisting of 12 males and 12 females (Table 1), were conducted. The mean age was 18.4 years. All the participants were christians because the study was conducted in a christian dominated area. All the participants were single, the majority (79%) are in a relationship but only (58%) reported engaging in sexual intercourse. Sixty-seven percent (67%) of the participants were raised in urban areas while 33% were raised in rural areas.

**Table 1:** sociodemographic characteristics and sexual behaviour of participants

S/N	University	Sex	Age	Where they grew up	Religion	Marital status	Relationship status	Ever had sex with someone
1	UNN	Male	19	Rural	Christianity	Single	Yes	Yes
2	UNN	Male	18	Urban	"	"	Yes	No
3	UNN	Male	19	Urban	"	"	No	No
4	UNN	Female	19	Urban	"	"	Yes	Yes
5	UNN	Female	18	Urban	"	"	No	Yes
6	UNN	Female	19	Urban	"	"	Yes	Yes
7	AE-FUNAI	Male	19	Urban	"	"	Yes	Yes
8	AE-FUNAI	Male	19	Urban	"	"	Yes	No
9	AE-FUNAI	Male	19	Urban	"	"	Yes	No
10	AE-FUNAI	Female	19	Rural	"	"	Yes	Yes
11	AE-FUNAI	Female	18	Urban	"	"	No	No
12	AE-FUNAI	Female	17	Urban	"	"	Yes	Yes
13	EBSU	Male	18	Urban	"	"	Yes	Yes
14	EBSU	Male	18	Rural	"	"	Yes	Yes
15	EBSU	Male	18	Urban	"	"	No	No
16	EBSU	Female	16	Rural	"	"	Yes	Yes
17	EBSU	Female	19	Urban	"	"	Yes	No
18	EBSU	Female	19	Rural	"	"	Yes	Yes
19	ESUT	Male	19	Rural	"	"	Yes	Yes
20	ESUT	Male	19	Rural	"	"	Yes	No
21	ESUT	Male	19	Rural	"	"	Yes	Yes
22	ESUT	Female	18	Urban	"	"	No	No
23	ESUT	Female	17	Urban	"	"	Yes	Yes
24	ESUT	Female	19	Urban	"	"	Yes	No

**Parents´ connectedness during early adolescence and risky sexual behaviour in the university:** the study found that parents´ presence throughout early adolescence means a lot for the child´s sexual experience in later phase of life. Adolescents who were connected with their parents during early adolescence reported that it influenced their sexual behaviour in the university. Closeness with parents offered them the opportunity to learn about the dangers of unprotected sex. The following quote from a female student is illustrative: “okay, somehow I found myself trying to do some things like sleeping with different guys, then I think about my parents, I remembered their advice, they taught me the disadvantages and about unprotected sex and how you can get STDs through sex. So because of that, I would not want to engage in those acts” [female student, AE-FUNAI]. Majority of the participants reported that parents do not prioritise sex education during early adolescence. However, unlike fathers, mothers are available to educate their children when the need arise. A male student captured it this way: “I was closer to my mom and I talked to her concerning some very important and secret things, and she hears me when she wants to. She doesn´t feel like it´s a must to listen to me but she does when she wants to. The last time I raised an issue about condoms, she did not treat it until weeks or months later and then she was like “ah! What happened?” I know she is very busy but not too busy that she won´t talk to me on very sensitive issues like having unprotected sex. But she is better, she is like a one-eyed man in the village of the blind, compared to my dad” [male student, UNN].

Participants also reported that risky sexual behaviours are noticed among adolescent students who were not properly educated about sex during early adolescence. They described “good parents” as those who impact comprehensive sex education on their children at early adolescence. An illustrative quote from one of the participants captured it this way: “for some people, their parents advised them, if you have a good parent, they would advise you not to engage in unprotected sex and that if you do, you might suffer the consequences. I was also told that initiating sexual intercourse too early in life is risky and has some grave consequences. So it is better to start at the proper time. I was also told not to sleep around with different people. So people that don´t have that kind of parents seem to engage in risky sex in the university” [male student, EBSU].

However, participants reported that adolescents who grew up around strict and authoritative parents engage more in risky sex in the university. This is because they see the university as a place to explore without parental interference. On that note, some of the participants indicated that parents should discuss contraceptives like condom with their children. One of the students said: “in a family where the parents are so strict and restrictive, kids tend to be curious because parents do not discuss things like condom use and sticking with one sexual partner. Even, at home, they are under all sorts of rules like, “don´t talk to the opposite sex, no trousers, cover your hair and don´t wear short skirts”. Immediately such children step out of their homes, they want to explore all sorts of things and do those things they were not allowed to do because their parents did not tell them all that they need to know” [female student, UNN]. Another participant, a female student added: “if parents can stop being so rigid and talk about sex with their children, I am not saying they should tell their children to have sex, no! I am saying that in as much as they teach their children to abstain from sex they should also teach the use of contraceptives like condoms. What happens in cases where they find themselves in situations that they can´t escape, get tempted or fall in love in the university? Having knowledge of and using contraceptives will help them avoid drug abuse and abortions” [female student, UNN].

**Religious experience during early adolescence and risky sexual behaviour in the university:** our findings showed that a link exists between the religious environment during early adolescence and risky sexual behaviour in the university. The restraining power of religion on risky sex was articulated in the following quote by a female student: “for someone like me who grew up in a christian home precisely Deeper Life, we don't engage in those behaviours (multiple sexual partners) because when we remember our background and the people surrounding us, we wouldn´t want them to question our character” [female student, ESUT]. Another female student added: “to a very large extent, morally I have learned that as an unmarried young lady I am supposed to keep myself and desist from indulging in sexual activities until I am married. But even if today I wish to do it, I will not start dating boys here and there, am I a prostitute?” [female student, AE-FUNAI].

However, our data showed that those raised in religious homes that did not provide sex education engage in risky sexual acts in the university. An illustrative quote from a female UNN student captured it this way: “some families are so religious to the extent that they don't talk about sex and when they do, they paint it as crime and evil. When their kids get into the university, they want to free themselves from the shackles or bondage of being over monitored and so they begin to explore all manner of sexual acts including the very risky ones” [female student, UNN]. Another participant, a female student added: “I still adhere to some of the religious teachings I learned from home in school but it does not stop me from doing whatever I want to do. Although I know it is a sin and within me, I know that what I am doing is not good but I just do it (have unprotected sex) to have fun and ease myself” [female student, EBSU].

**Peer group during early adolescence and risky sexual behaviour in the university:** our findings show that connectedness with peer group during early adolescence does not contribute to risky sexual acts in the university. Nevertheless, peer groups during early adolescence served as source of knowledge on sexual activities as shown in the following quote from a female student: “before I gained admission, there were things about sex that I learned from my friends” [female student, AE-FUNAI]. Although, peer group during early adolescence often encourage adolescents to have multiple sexual partners when they enter the university, as illustrated by a male student in AE-FUNAI: “they will tell you that since your girlfriend is not close (not in this school) that you don't know how she is living her life; you should therefore try another girl” [male student, AE-FUNAI]. However, our data showed that adolescents who reported having multiple sexual partners insist that it is not influenced by the association with peers during early adolescence. A male UNN student captured it this way: “no, it was a personal decision, I just wanted to try something new” [male student, UNN]. Another participant added: “it is based on my decision, if I choose to have unprotected sex, it would not be because my friends convinced me to. That´s just it! It´s just a matter of personal decision” [female student, UNN].

**Place of residence during early adolescence and risky sexual behaviour in the university:** finally, the study found that the social environment during early adolescence also influenced adolescents´ sexual behaviour in the university. Unlike adolescents raised in urban areas, those who spent their early adolescence in rural areas were reported to be involved in risky sexual acts in the university because they were not properly guided. One of the students raised in rural area said: “those who were raised in the village are naïve. Their parents don't talk about sex and when they do, they give you reasons not to get involved in sexual activity and not about the use of contraceptives or condoms. So when these students are admitted into the university, they are less likely to use them because they depend on what they learned from friends, the internet, or biology class, some even use counting method or withdrawal which may not be reliable” [female student, AE-FUNAI]. Another male student added: “many young people engage in unprotected sex especially those from rural areas because they are not aware of the consequences” [male student, UNN].

Exposure to sex education increased the likelihood of safe sex and condom use among urbanites, but for adolescents who spent their early adolescence in rural areas, misinformation and superstitious beliefs about contraceptives decreased usage. One of the participants, a male student said: “those that come from the urban areas have a better knowledge of safe sex. In the rural areas - I think they are misinformed or uninformed, they do not know much about safe sex and the few that know do not know how it works and so to a large extent, I feel that urbanites know better about sex because they have had sex education at one point. In the rural areas, there are some kind of superstitious beliefs about contraceptives so the majority do not use it” [male student, ESUT].

## Discussion

We explored how social context of early adolescence influenced adolescents´ risky sexual behaviour in the university. We found that parents´ presence during early adolescence mean a lot for their sexual behaviour in later life. Parents' presence manifests in terms of connectedness and availability to educate them about sex. In our findings, sex education featured strongly as an important asset for shunning risky sex in later phase. Sex education by parents during early adolescence empowered adolescent students to take charge of their sexuality by avoiding sexual activities that would affect their health in the future. This finding agrees with that of Hutchison and Cooney [23] which is that when parents provide sex education it leads to more effective contraceptive use at later phase. Manyike *et al*. [24] similarly found that children educated by their parents about sex were less likely to be sexually abused than those not educated by their parents. It is important to note that our emphasis on sex education by parents rather than by school is because parents have the primary role of educating their children about sex, especially at early development [25]. Although adolescents can get sex information anywhere (i.e. school, media, books, friends, etc.), it is the duty of parents to regulate and review each information especially at the earlier stage of life. In fact, they could withdraw their children from school if they are not comfortable with the pattern of sex education [26].

The study also found that adolescents' risky sexual behaviours in the university are shaped by the place they were raised during early adolescence. Specifically, adolescents who were raised in urban areas were reported to be better informed about safe sex. A similar finding was reported by Adeboyejo and Onyeonoru [27] in their study of adolescents´ sexual behaviours in South-west Nigeria. They concluded that urbanites are better exposed to technologies and other materials to learn about contraceptives like condoms. Emerging data revealed that adolescents who grew up in rural areas do not have comprehensive information about sex and contraceptives and this increased risky sexual behaviour in the university environment. Also, misinformation and superstitious beliefs were found to hinder contraceptive use by those who were raised in rural areas. Atama *et al*. [28] similarly found, in their study in Benue state, that belief in the community deity hinders contraceptive use among women.

Despite the importance of sex education, we found that parents, especially fathers, are reluctant to educate their children about safe sex during early adolescence. This was also reported by Hutchison and Cooney [23]. However, sex education by both parents would likely increase impact. Emerging data also showed that when parents maintain strict and authoritative parenting style that does not include comprehensive sex education, adolescents tend to engage in risky sexual acts in the university. This disagrees with the findings of Yimer and Ashebir [29], which reported that authoritative parenting, is associated with decreased odds of engaging in risky sexual behaviour. In this study, participants from authoritative homes see the university environment as a place to do what they were prevented from doing at home, which includes exploring their sexuality. This suggests that unprotected sexual intercourse and multiple sexual partners in the university are linked to low parent connectedness and non-exposure to comprehensive sex education during early adolescence. Abstinence-only sex education has been associated with unprotected sex, thereby increasing the risk of unwanted pregnancies and STIs [19].

Religious beliefs during early adolescence may serve as a protective factor in sexual initiation as well as contraceptive use and multiple sexual partners in the university. Adolescents from strong religious background recounted that they are restrained from engaging in sexual acts that would dishonour them and their family. These included having multiple sexual partners and having unprotected sex that increases the risk of STIs and unwanted pregnancy. The findings agree with those of Wosu [30] and Burdette and Hill [31], that high religiosity makes adolescents avoid premarital sex despite the urge. Our study also showed that having a strong religious background would not always influence safer sex or abstinence, what counts, therefore, is parents´ ability to teach comprehensive sex education alongside religious principles and morals.

Unlike other studies, we found that connectedness to peer groups during early adolescence did not influence the likelihood of risky sexual behaviour in the University. For instance, Bingenheimer *et al.*[32] found that peer association was linked to sexual initiation. In our study, we found that social relationships with peers at early adolescence only served as source of information on sexual activities but did not contribute to risky sexual behaviour like having multiple sex partners at later adolescence. This position was supported by UNICEF [17], that late adolescence is a phase where the opinion of peer group starts diminishing as adolescents gain more confidence and clarity in their own identity and opinions. Keizer, Helmerhorst and Gelderen [33] found that although adolescents spend more time with their peers, the attachment with parents appear to be more important for assessing how adolescents behave.

Our study has shown that early adolescence is an important developmental phase. Consistent with the DCF, perpetration of risky sexual behaviours by adolescents in the university was associated with the social context of early adolescence. Besides peer groups, activities and interactions with parents, religious values and community of residence at early adolescence forms the basis for risky sexual behaviours like unprotected sex and multiple sexual partners in the university. Examining sex education at this stage provides clear understanding of how each factor contributed to adolescents´ risky sexual behaviour in the university. Though parents may not be present throughout adolescents´ lives [34], impacting the right sex education will greatly shape how adolescents practice safe sex in the future.

Our study is limited on the basis that it focused only on proximal microsystems (e.g. peer group, parents, religious group and community of residence) and not distal macro systems like culture and society. Likewise, contextual factors within university environment and gender differences were not explored. Nevertheless, our study has successfully shown that key contexts like parents, religious religion and community of residence are important resources for adolescents´ healthy development.

## Conclusion

The social context of early adolescence is important to our understanding of risky sexual behaviours in the university. Adolescents who were raised in an environment that provided comprehensive sex education tend to abstain or practice safer sex at the university. The role of parents cannot be overemphasized because they mould adolescent´s behaviour starting from early stages of development to later phase of development. Parents are therefore implored to educate their children about sex to reduce risky sexual behaviours as they grow older.

### What is known about this topic

- Adolescent students tend to be liberal in their sexual attitudes and behaviours;
- Adolescents in the university engage in risky sexual behaviours like unprotected sex and multiple sexual partners;
- Social context shapes people´s daily experiences and also, directly and indirectly, affect their behaviour.

### What this study adds

- Sex education acquired from parents during early adolescence reduces the chances of adolescent students engaging in risky sexual behaviour in the university;
- Religious values, acquired during early adolescence, may not reduce the indulgence in risky sexual acts except when it is combined with comprehensive sex education;
- Peer group connectedness during early adolescence does not influence adolescents´ risky sexual behaviours in the university.
